# β-glucans from *Agaricus bisporus* mushroom products drive Trained Immunity

**DOI:** 10.3389/fnut.2024.1346706

**Published:** 2024-02-15

**Authors:** Sarah Case, Tara O'Brien, Anna E. Ledwith, Shilong Chen, Cian J. H. Horneck Johnston, Emer E. Hackett, Michele O'Sullivan, Hugo Charles-Messance, Elaine Dempsey, Supriya Yadav, Jude Wilson, Sinead C. Corr, Shipra Nagar, Frederick J. Sheedy

**Affiliations:** ^1^School of Biochemistry and Immunology, Trinity College, Dublin, Ireland; ^2^NatPro Centre, School of Pharmacy and Pharmaceutical Sciences, Trinity College, Dublin, Ireland; ^3^School of Genetics and Microbiology, Trinity College, Dublin, Ireland; ^4^MBio, Monaghan, Ireland; ^5^APC Microbiome Ireland, University College Cork, Cork, Ireland

**Keywords:** mushroom, Trained Immunity, β-glucan, digestion, immunometabolism

## Abstract

**Introduction:**

Macrofungi, such as edible mushrooms, have been used as a valuable medical resource for millennia as a result of their antibacterial and immuno-modulatory components. Mushrooms contain dietary fibers known as β-glucans, a class of polysaccharides previously linked to the induction of Trained Immunity. However, little is known about the ability of mushroom-derived β-glucans to induce Trained Immunity.

**Methods & results:**

Using various powdered forms of the white button mushroom (Agaricus bisporus), we found that mouse macrophages pre-treated with whole mushroom powder (WMP) displayed enhanced responses to restimulation with TLR ligands, being particularly sensitive to Toll-like receptor (TLR)-2 stimulation using synthetic lipopeptides. This trained response was modest compared to training observed with yeast-derived β-glucans and correlated with the amount of available β-glucans in the WMP. Enriching for β-glucans content using either a simulated *in-vitro* digestion or chemical fractionation retained and boosted the trained response with WMP, respectively. Importantly, both WMP and digested-WMP preparations retained β-glucans as identified by nuclear magnetic resonance analysis and both displayed the capacity to train human monocytes and enhanced responses to restimulation. To determine if dietary incorporation of mushroom products can lead to Trained Immunity in myeloid cells in vivo, mice were given a regimen of WMP by oral gavage prior to sacrifice. Flow cytometric analysis of bone-marrow progenitors indicated alterations in hematopoietic stem and progenitor cells population dynamics, with shift toward myeloid-committed multi-potent progenitor cells. Mature bone marrow-derived macrophages derived from these mice displayed enhanced responses to restimulation, again particularly sensitive to TLR2.

**Discussion:**

Taken together, these data demonstrate that β-glucans from common macrofungi can train innate immune cells and could point to novel ways of delivering bio-available β-glucans for education of the innate immune system.

## Introduction

Memory-like properties have recently been ascribed to cells of the innate immune system (1). The mechanisms underlying these vary with cell type; however, triggering enhanced responsiveness in myeloid cells including macrophages and monocytes has been termed Trained Immunity (2). Exposure to specific Training stimuli leads to functional reprogramming in these cells and their hematopoietic progenitors, which includes metabolic reprogramming and epigenetic priming of inflammatory genes. This trained response is non-specific, meaning trained cells respond more efficiently to a broad range of stimuli which re-activate them (2). Trained Immunity has been proposed to underlie protective effects like heterologous immunity seen in some vaccines and increased resistance to infection, by promoting innate immune function. However, it has also been implicated in various inflammatory diseases where metabolic and disease-associated stimuli train innate immune cells for inappropriate activation underlying pathogenesis (2, 3). Recent evidence suggests that the long-term protective effects of Trained Immunity proceed not through altering the function of peripheral cells like monocytes and macrophages, but rather through altering the fate of central medullary hematopoietic stem-progenitor cells HSPCs, which give rise to mature myeloid cells (2, 4).

One of the earliest training stimuli described were fungal β-glucans (5), structural carbohydrates found in cell walls, which trigger the innate immune receptor Dectin-1 (6, 7). β-glucans are a diverse family of biomacromolecules found across multiple kingdoms of life, which differ in their physical and chemical properties and their interactions with mammalian cells (8, 9). Signaling through Dectin-1 is a key step for the initiation of β-glucan-induced Trained Immunity (5), although fungal β-glucans can trigger other receptors, including CR3 on neutrophils to prime for degranulation in a complement-dependent manner (10), and can co-operate with CR3 to drive pro-inflammatory cytokine IL-1β production and cell death responses in macrophages (11). Crucially, differences in Dectin-1 binding and responses have been described between low MW, soluble β-glucans and larger, particulate β-glucans. Goodridge et al., reported that particulate β-glucans drive the phagocytotic synapse for full anti-microbial activity in myeloid cells, which can be blocked by binding of Dectin-1 by low MW β-glucans (12). Different Dectin-1 isoforms exist which differ in the length of the stalk region. Low MW, soluble β-glucans seem to bind and signal through the less abundant but larger isoform, Dectin-1a, while particulate β-glucans can bind through both isoforms (13, 14). Many of the processes involved in β-glucan driven Trained Immunity have been reported using a β(1→3)-glucan preparation from *Candida albicans* (5, 15–18), although evidence is emerging that baker's yeast *Saccharomyces cerevisiae*-derived β-glucans can also drive this process (19–23). The impact of more common β-glucans on Trained Immunity, particularly those found in foods, like plant/oat and mushroom β-glucans, is less well described (24, 25).

Edible mushrooms represent a diverse class of macrofungi and apart from their nutritional benefits, have long been held as sources of novel medicinal and psychoactive compounds (26). Outside the mushroom cell membrane, β-glucans lie between an inner layer of chitin and outer layer of mannoproteins, the latter of which have been described to have anti-inflammatory properties (27). β-glucans in mushrooms are generally more soluble due to shorter, more linear glucose polymers made up of β(1→3) and/or β(1→6) glycosidic linkages, with less branching than β-glucans from other fungal species like yeasts (28, 29). They can further be differentiated structurally from β-glucans present in oat and cereals, which comprise of β(1→3, 1→4)-glucose units (30). Laminarin from brown seaweed represents another category of β-glucans composed of β(1→3) and β(1→6) linkages, however is distinguishable from mushroom β-glucans due to differences in their glucose-linked backbone. Laminarin has a backbone of β(1→3) with branching at C6, which is converse to mushroom β-glucans encompassing a β(1→6) linked backbone with some branching at C3 and occasionally at C4 (31–33). Because of these chemical differences, it has been speculated that mushroom β-glucans are more immunologically inert and hence, well tolerated by humans. Despite this, β-glucans from edible mushrooms have been promoted for immune benefits in a variety of settings worldwide and there is considerable evidence that mushroom-derived β-glucans and other compounds enhance/augment anti-cancer therapies (26, 34). We thus hypothesize that mushroom β-glucans may support innate immune function through triggering innate immune memory responses via Trained Immunity. To test this, we used powdered mushroom derived from the common white button mushroom species, *Agaricus bisporus* (35) and tested the capacity of this to trigger Trained Immunity using a number of established assays (18, 23). We also included *Saccharomyces cerevisiae*-derived whole glucan particles (WGP) and Laminarin in the study to compare the performance and efficacy of β-glucans from different sources.

## Results

### Mushroom powders contain modest concentrations of β-glucan with distinct Dectin-1 binding properties

**Figure 1:**
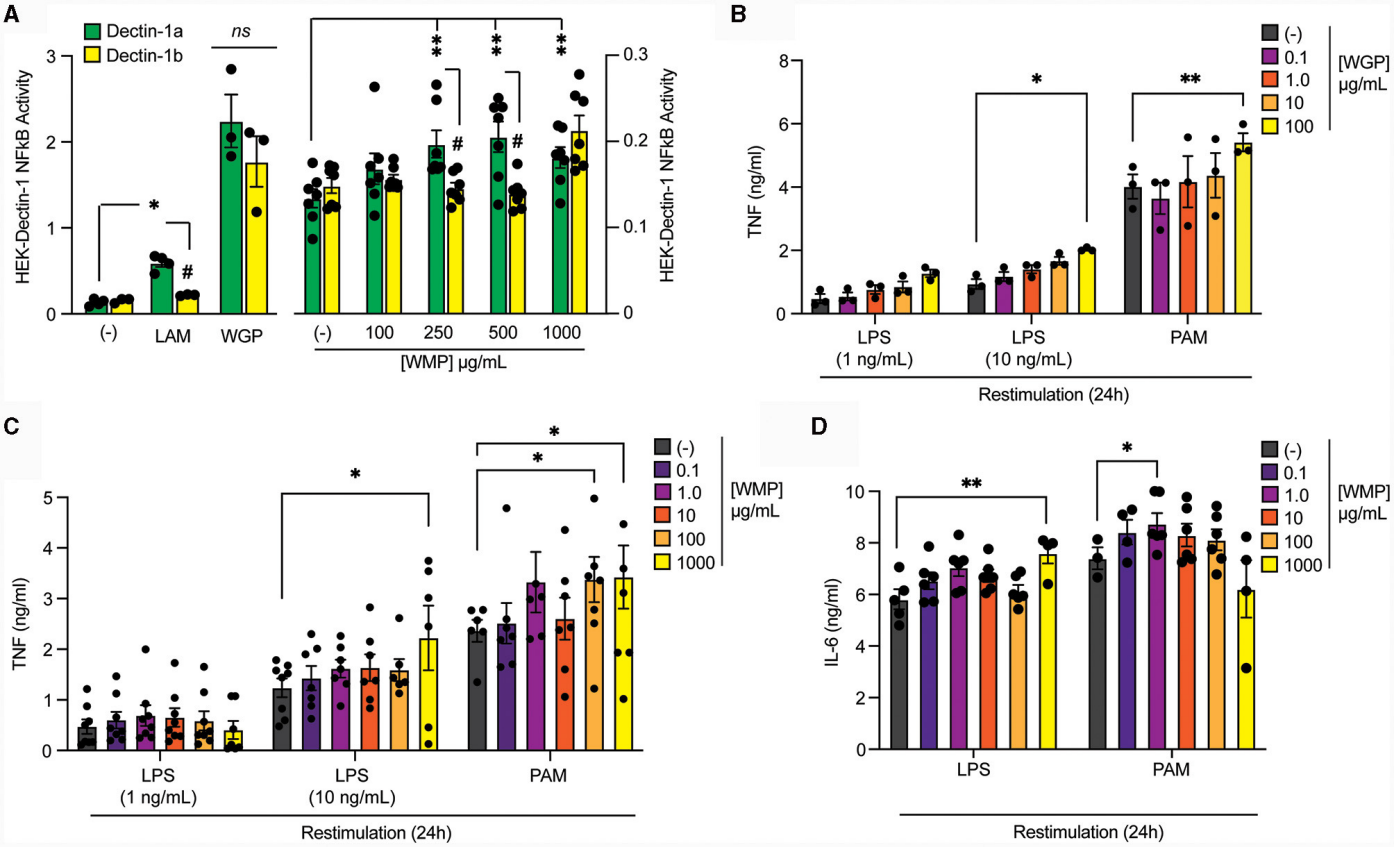
Pre-treatment of macrophages with mushroom powders augments long-term responses to restimulation. **(A)** HEK-Dectin-1a or HEK-Dectin-1b-cell lines were treated with the increasing concentrations WMP (0.1–1,000 μg/mL), laminarin (1 μg/mL), WGP (10 μg/mL) or left untreated (-) for 24 h. SEAP activity was measured using QuantiBlue. Data is mean relative induction over untreated ± s.e.m. of *n* = 3–6 replicates. **(B–D)** Mature bone-marrow derived macrophages (BMDMs) were treated with yeast-derived whole-glucan particle [WGP, **(B)**], *A. bisporus* derived whole mushroom powder [WMP, **(C, D)**] at the indicated concentrations (μg/mL) or left untreated (-). After 24 h, media was removed and cells washed and matured for a further 7 days prior to restimulation with LPS (at indicated concentrations or 10 ng/mL) or Pam3CSK4 (PAM, 10 μg/mL) for 24 h. Supernatant was removed and the indicated cytokines measured by ELISA [TNF, **(B, C)** & IL6, **(D)**]. Data is mean cytokine concentration ± s.e.m. of *n* = 3 **(B)**, *n* = 6 **(C, D)** independent experiments. */^#^*P* value < 0.05, **P value < 0.01, determined using 2-way ANOVA with the indicated *post-hoc* multiple comparisons (Fisher's LSD test).

Since fungal compounds have been linked to the induction of Trained Immunity in myeloid cells, we investigated if the powdered form of edible white button mushroom (*A. bisporus*) could induce similar effects in macrophages. We previously demonstrated that these unprocessed powders contain ~8% β-glucan by dry weight [(35) and Table 1]. This compares to 70%−80% in other commercially available and concentrated yeast-derived β-glucan purifications, notably *Saccharomyces cerevisiae*-derived whole glucan particles (WGP) [(28) and Table 1]. We began by examining the ability of various β-glucan containing preparations to bind and signal through the Dectin-1 receptor using *in-vitro* reporter assays, specifically HEK293-cells overexpressing either the Dectin-1b or Dectin-1a isoform. Ten μg/mL of yeast WGP triggers activation of an NFκB-linked reporter gene to a similar extent across both cell types, while Laminarin [a macroalgal-derived low MW β-glucan (36)] drives Dectin-1a only (Figure 1A left panel). Given the differences in % β-glucan content in WGP and WMP, we tested a range of WMP concentrations in HEK-Dectin-1a/b cells—with 100 μg/mL of WMP containing equivalent total β-glucan content to 10 μg/mL of WGP. WMP drove a dose-dependent response in HEK-Dectin1a cells only and to a much lower extent than seen with Laminarin or WGP, despite controlling for differences in β-glucan content by testing a higher range of concentrations (250–1,000 μg/mL, Figure 1B right panel). The increased binding to Dectin-1a suggest that the β-glucans contained in WMP are structurally different to yeast β-glucan and likely represent more soluble, less branched β-glucans (29) (akin to Laminarin). Since matching for total β-glucan concentrations could not drive similar levels of NFκB-linked Dectin-1 signaling, it is possible that WMP contains polysaccharides (e.g., a higher relative ratio of α-glucans, Table 1) and other molecules that interfere with Dectin-1 binding. Having thus measured activation of Dectin-1a by WMP, we proceeded to assess the impact of these WMPs on innate immune memory responses.

**Table 1:** Megazyme assay glucan content of dry powders/preparations.

**Sample**	**[Total Glucan] w/w (mean ±s.d.)**	**[α-Glucan] w/w (mean ±s.d.)**	**[β-Glucan] w/w (mean ±s.d.)**
Barley preparation^*^	43.9 ± 0.6	0.4 ± 0.2	43.5 ± 0.7
Yeast WGP	78.5 ± 4.5	5.5 ± 0.8	73.0 ± 4.2
Filtered WMP	10.2 ± 0.1	1.2 ± 0.1	8.9 ± 0.1
Se-WMP	12.4 ± 0.3	5.0 ± 0.2	7.4 ± 0.5
VitD-WMP	8.7 ± 0.6	1.1 ± 0.2	7.6 ± 0.6
IVD-WMP	14.5 ± 0.3	1.0 ± 0.0	13.5 ± 0.3

^*^Barley preparation used as positive control for Megazyme assay.

### Pre-treatment of macrophages with mushroom powders augments long-term responses to restimulation

To compare the ability of these different β-glucans to impact Trained Immunity, we exposed mature mouse bone-marrow derived macrophages (BMDMs) to the β-glucan-containing compounds for 24 h followed by an extensive wash-out phase. Cells were further matured for an additional 7-days before restimulating with TLR ligands, either LPS (TLR4) or Pam3CSK4 (PAM, TLR2). Significantly enhanced TNF production was observed in cells trained with a high concentration of yeast-derived WGP (100 μg/mL) for both LPS and PAM restimulation (Figure 1B). Training BMDM with similar concentrations of WMP (between 0.1–1,000 μg/mL) leads to enhanced TNF responses to LPS and PAM restimulation (Figure 1C), to a similar extent as that seen with an equivalent β-glucan concentration as WGP (1,000 μg/mL WMP; ~1.5–2.0-fold over untrained controls), albeit with more variation. Interestingly, PAM restimulation is more sensitive to training with WMP than LPS, with enhanced responses observed at lower concentrations of WMP (100–1,000 μg/mL). A similar effect was observed when the production of the pro-inflammatory cytokine IL6 was measured (Figure 1D). Thus, training with WMP can drive similar enhanced long-term responses to restimulation to that seen with equivalent concentrations of yeast-derived β-glucans, albeit with more variation to that observed with more pure preparations.

### Vitamin-D2 enriched mushroom powder retains the capacity to train macrophages

**Figure 2:**
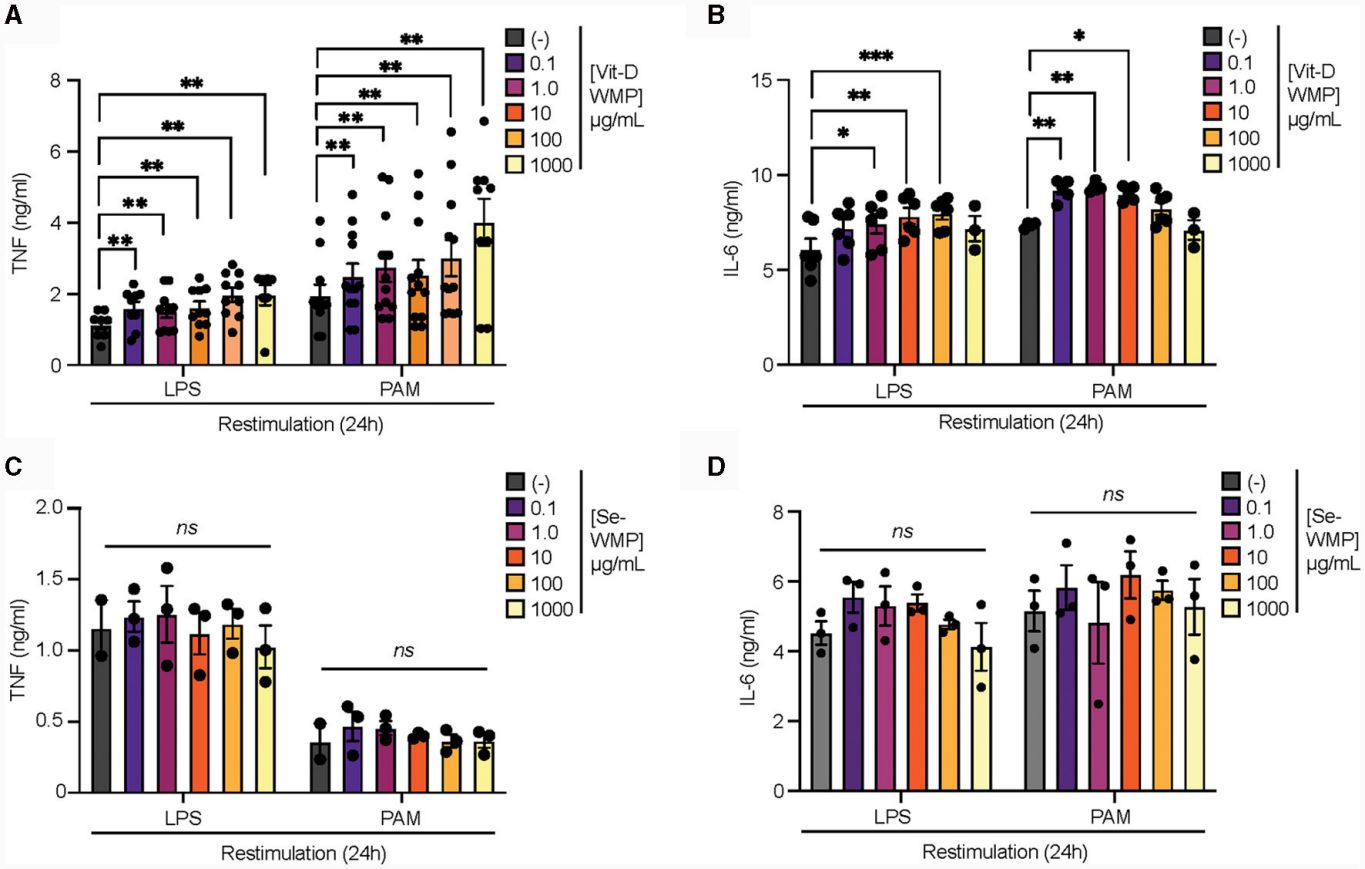
Vit-D enriched mushroom powder retains the capacity to train macrophages. **(A–D)** Mature BMDMs were treated with Vitamin-D (Vit-D) enriched or Selenium (Se-) enriched *A. bisporus* derived whole mushroom powder (WMP) at the indicated concentrations (μg/mL) or left untreated (-). After 24 h, media was removed and cells washed and matured for a further 7 days prior to restimulation with LPS (10 ng/mL) or Pam3CSK4 (PAM, 10 ug/mL) for 24 h. Supernatant was removed and the indicated cytokines measured by ELISA [TNF, **(A, C)** & IL6, **(B, D)**]. Data is mean cytokine concentration ± s.e.m. of *n* = 8 **(A)**, *n* = 6 **(B)** or *n* = 3 **(C, D)** independent experiments. *P value < 0.05, **P value < 0.01, ^***^P value < 0.005, determined using 2-way Mixed Effect ANOVA with the indicated *post-hoc* multiple comparisons (Fisher's LSD test).

Edible mushrooms naturally enriched in micronutrients are being developed to increase bioavailability (35, 37, 38). However, their impact on immune function is unclear. Here, we tested the ability of Vit-D(2) enriched WMP and high Selenium-WMP to impact Trained Immunity in BMDM. Vit-D WMP drove similar responses as previously observed with WMP, particularly enhanced TNF responses to PAM restimulation, even at doses as low as 1 μg/mL (Figure 2A). Similarly, both LPS and PAM responses were significantly enhanced with Vit-D WMP when IL6 production was measured (Figure 2B). Selenium has been linked to anti-inflammatory responses in other cases (35), and accordingly, we did not observe significant Trained responses in BMDM trained with Se-enriched WMP when TNF or IL6 production was measured (Figures 2C, D). Although overall β-glucan concentration is not significantly altered in Se-enriched WMP relative to regular WMP, an increase in α-glucans was measured specifically in Se-enriched WMP (Table 1), which may alter immune training activity.

### Mushroom β-glucans drive enhanced responses to restimulation

**Figure 3:**
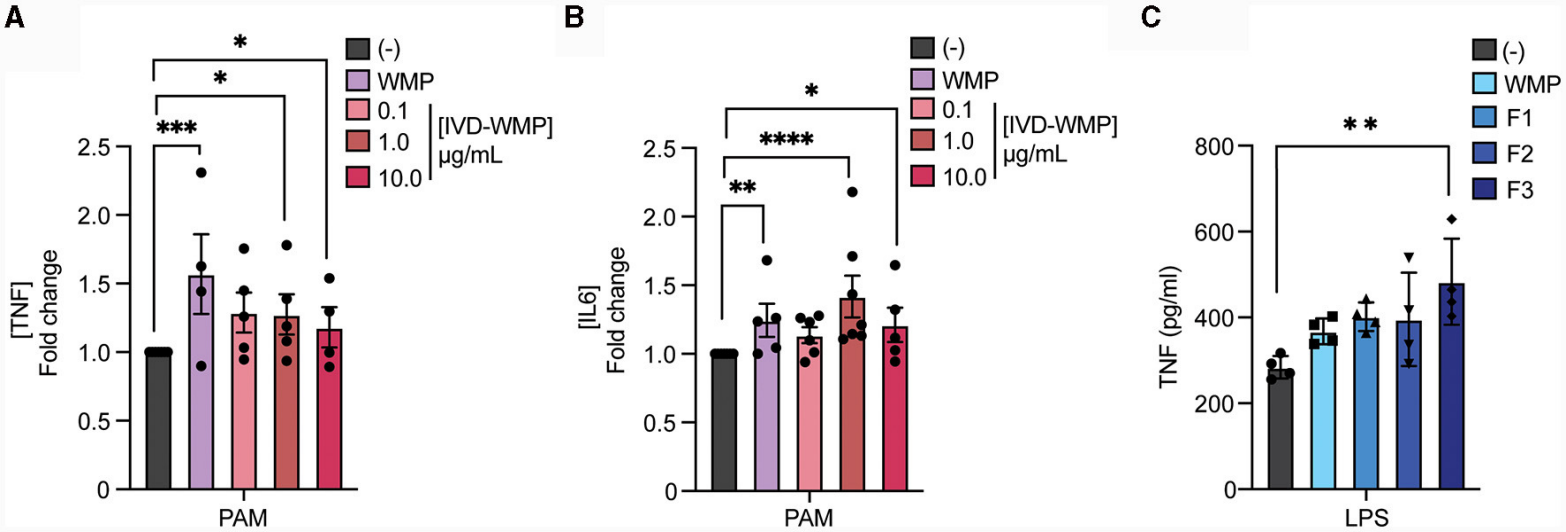
Mushroom β-glucans drive enhanced responses to restimulation. **(A, B)** Mature BMDMs were treated with the undigested product of simulated *in-vitro* digestion of *A. bisporus* derived whole mushroom powder (IVD-WMP) at the indicated concentrations (μg/mL), undigested WMP (100 μg/mL) or left untreated (-). After 24 h, media was removed and cells washed and matured for a further 7 days prior to restimulation with Pam3CSK4 (PAM, 10 ug/mL) for 24 h. Supernatant was removed and the indicated cytokines measured by ELISA [**(A)**, TNF & **(B)**, IL6]. Data is mean fold change in cytokine production normalized to untreated cells (-) ± s.e.m. of *n* = 5 independent experiments. **(C)** Mature BMDMs were treated with glucan-enriched fractions from *A. bisporus* derived whole mushroom powder (F1-F3, 100 μg/mL), unfractionated WMP (100 μg/mL) or left untreated (-). After 24 h, media was removed and cells washed and matured for a further 7 days prior to restimulation with LPS (10 ng/mL) for 24 h. Supernatant was removed and TNF measured by ELISA. Data is mean cytokine concentration ± s.e.m. of *n* = 3 independent experiments. *P value < 0.05, **P value < 0.01, ^***^P value < 0.005, ^****^ *P* value < 0.001 determined using 1-way ANOVA with the indicated *post-hoc* multiple comparisons (Sidak's multiple comparisons test).

Our data thus far suggests that WMP contains the capacity to train myeloid cells in the long-term. However, whether consumption of mushrooms orally drives similar responses *in vivo* is unclear. To begin to address this, we performed simulated *in-vitro* digestion of WMP (39) and examined the impact of the undigested products on BMDM function. As previously reported (35), the simulated digestion process retains and slightly enriches total β-glucan content of WMP, increasing to ~13% in *in-vitro* digested product of WMP (IVD-WMP, Tables 1, 2). We found that 3 concentrations of IVD-WMP could drive trained responses over control untrained BMDM. However, this was not concentration-dependent and did not match the training seen with a high concentration of WMP (100 μg/mL, Figures 3A, B). Aside from enriching β-glucan content (as measured by Megazyme assay, Table 1), the simulated digestion also alters total carbohydrate concentration (40) (Table 2, going from 6.2 ± 0.6% to 17.6 ± 1.4% w/w) and therefore likely alters the structure and composition and activity of digestion-resistant fiber in the digested product, making direct comparisons to undigested powder more difficult. Despite this, significant enhancement of both TNF and IL6 responses are observed in IVD-WMP trained cells. The alterations in β-glucan content suggest that β-glucan is indeed the active component driving Trained Immunity by WMP. To test this more formally, we analyzed the Trained Immunity properties of 3 fractions of *A. bisporus*, ranging from hot-water extracted (F1) to 2 more basic wash fractions (F2; KOH & F3, NaOH), which should be more enriched in β-glucans than regular water soluble WMP (41). Consistent with this, we observed enhanced responses to LPS restimulation, particularly significant in the F3-trained BMDM (Figure 3C). These data suggest that β-glucan is the bioactive component of WMP, which drives Trained Immunity responses in macrophages.

**Table 2:** Effect of filtration and simulated digestion on total carbohydrate content of powders.

**[Carbohydrate Content], % w/w**			
		**Mean**	**SD**
WMP	Unfiltered	20.8	1.9
	Filtered	6.2	0.6
IVD-WMP	Unfiltered	48.2	2.2
	Filtered	17.6	1.4

### Mushroom products train human monocytes

**Figure 4:**
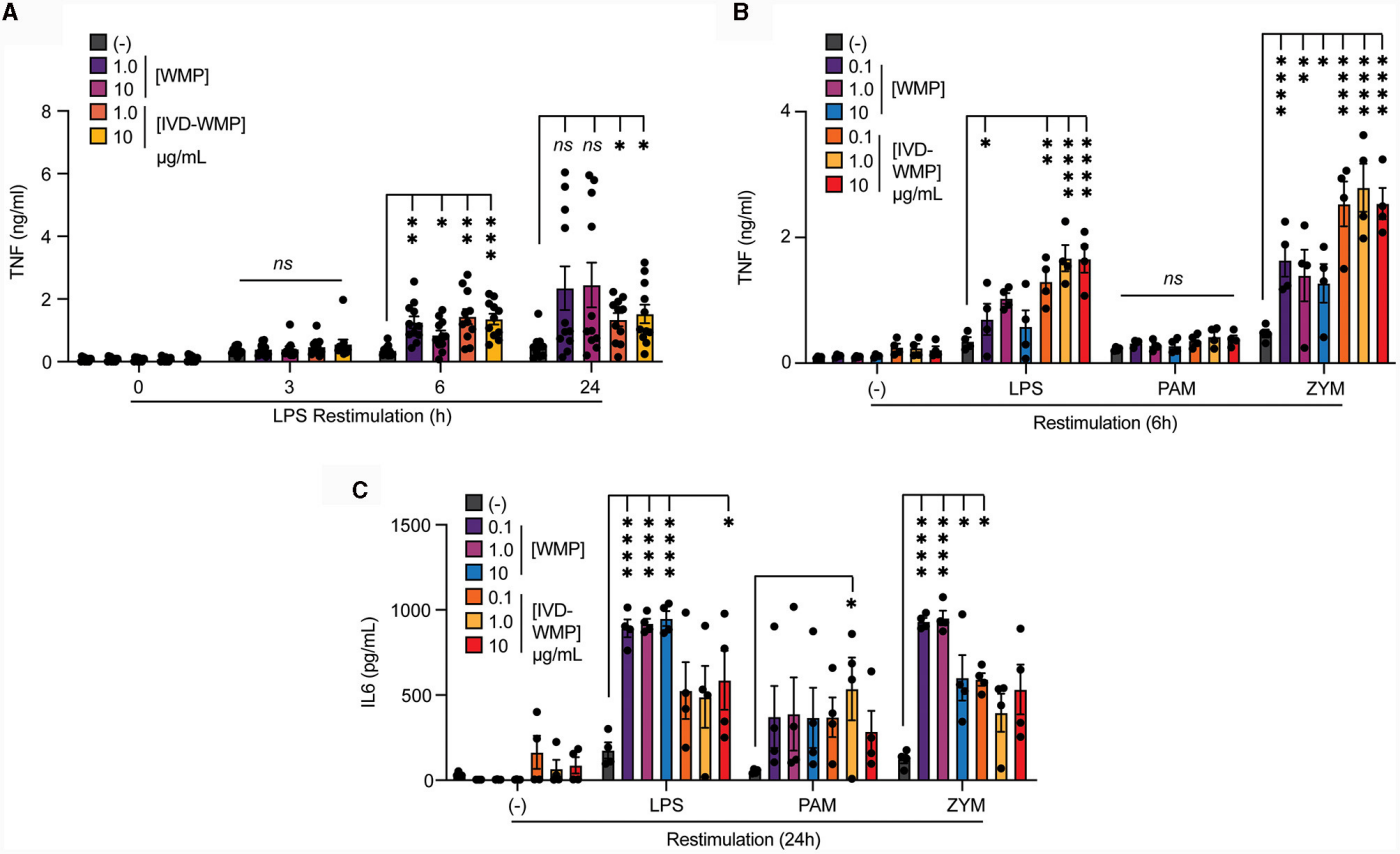
Mushroom products train human monocytes. **(A–C)** Monocytes from human PBMCs were isolated and treated with *A. bisporus* derived WMP or the undigested product of simulated *in-vitro* digestion of *A. bisporus* derived whole mushroom powder (IVD-WMP) at the indicated concentrations (μg/mL), or left untreated (-). After 24 h, media was removed and cells washed and matured for a further 6 days prior to restimulation with LPS (10 ng/mL) for the indicated times between 0–24 h **(A)**, or restimulated with LPS (10 ng/mL), (PAM, 10 μg/mL), Zymosan-A (ZYM, 10 μg/mL) or left untreated (-). TNF production was measured after 6 h **(B)** or IL6 production measured after 24 h **(C)**. Data is mean cytokine concentration ± s.e.m. of *n* = 11 **(A)** or *n* = 4 **(B, C)** donors. *P value < 0.05, **P value < 0.01, ^***^P value < 0.005, ^****^P value < 0.001 determined using 2-way ANOVA with the indicated *post-hoc* multiple comparisons (Fisher's LSD test).

To determine if human cells can be modulated by WMP in a similar way, we isolated monocytes from human blood and incubated these with WMP or similar bulk concentrations of the *in-vitro* digested powder (IVD-WMP) for 24 h as before. After washing, cells are matured to human-monocyte derived macrophages (hMDM) for a further 6-days before restimulation (18). We first examined the kinetics of enhanced TNF production after restimulation with LPS (Figure 4A). Consistent with Trained Immunity altering kinetics of inflammatory and immune genes (2), we observed significantly enhanced TNF production as early as 6h post-stimulation with both 2 concentrations of regular WMP and similar doses of IVD-WMP. This was maintained at 24 h in IVD-WMP trained hMDM. Using a wider panel of restimulation signals, we observed enhanced TNF production to LPS and the Dectin-1 fungal ligand Zymosan-A (ZYM). In contrast to BMDMs, responses to TLR2 stimulation via PAM were not significantly enhanced in the hMDM system (Figure 4B). Beyond TNF, IL6 production was also significantly enhanced in response to LPS, PAM and ZYM restimulation (Figure 4C). These results are consistent with the idea that Trained Immunity has broad non-specific responses in human cells (2, 42).

### Mushroom products drive metabolic reprogramming in trained myeloid cells

**Figure 5:**
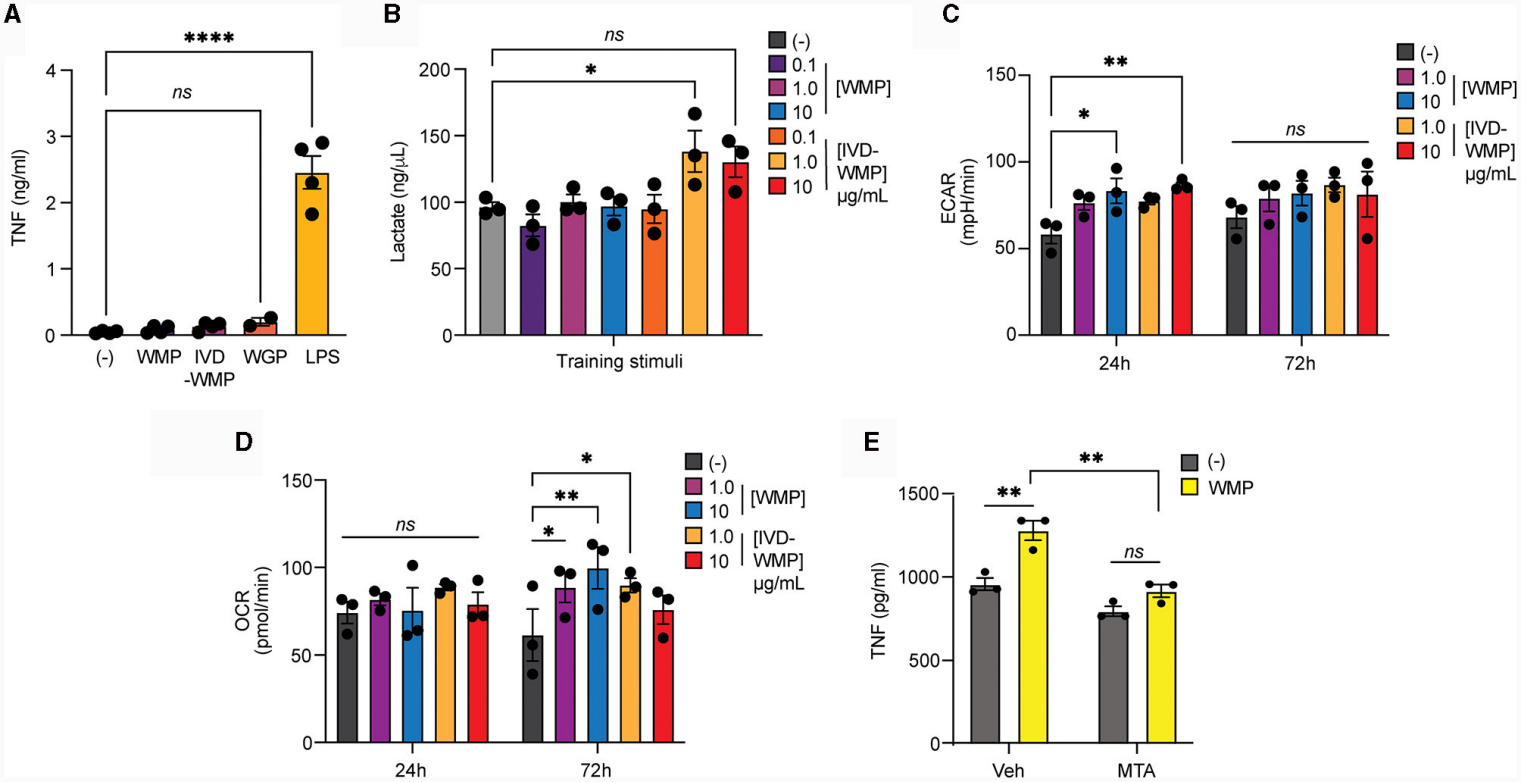
Mushroom products drive metabolic reprogramming in myeloid cells. **(A, B)** Monocytes from human PBMCs were isolated and treated with *A. bisporus* derived WMP or the undigested product of simulated *in-vitro* digestion of *A. bisporus* derived whole mushroom powder (IVD-WMP) at the indicated concentrations (μg/mL) or left untreated (-). Extracellular TNF production **(A)** or Lactate accumulation **(B)** was measured after 24h treatment. Data is mean concentration ± s.e.m. of *n* = 4 donors. **(C, D)** Mature BMDM were stimulated with WMP or IVD-WMP as indicated or left untreated (-) and metabolic flux measured on distinct Seahorse plates at 24 h or 72 h. Data is the calculated mean basal extracellular acidification rate [ECAR, **(C)**] or oxygen consumption rate [OCR, **(D)**] for *n* = 3 replicates. **(E)** Mature BMDM were treated with 5-methyladenosine (MTA, 2 mM) or vehicle control for 1 h prior to training with 1,000 μg/mL WMP. After 24 h, media was removed and cells washed and matured for a further 7 days prior to restimulation with Pam3CSK4 (PAM, 10 μg/mL) for 24 h. Supernatant was removed and TNF production measured by ELISA. Data is mean TNF concentration ± s.e.m. of *n* = 3 independent experiments. *P value < 0.05, **P value < 0.01, ^****^P value < 0.001, determined using 2-way ANOVA with the indicated *post-hoc* multiple comparisons (Fisher's LSD test).

Metabolic reprogramming has emerged as a hallmark of Trained Immunity (15). Rapid phagocytosis of β-glucan limits inflammatory activation (43) and we recently demonstrated that this process also promotes intracellular reprogramming required for trained responses (*in-press*). We now demonstrate that acute stimulation of mouse BMDM with mushroom powders or digested products triggers minimal TNF production [Figure 5A; and (35)], similar to yeast WGP. Whereas LPS, which drives long-term tolerance (44), triggers significant TNF production. We thus examined Lactate production as a surrogate of glycolytic activity in human monocytes after mushroom powder treatment. We detected significant up-regulation of extracellular Lactate production only after stimulation with a high concentration of IVD-WMP (Figure 5B). We thus further analyzed metabolic responses using the more sensitive extracellular flux analysis system in BMDM. Extracellular acidification rate (ECAR) was found to be up-regulated in BMDM after 24 h treatment with higher concentrations (10 μg/mL) of both WMP and IVD-WMP with increased basal ECAR (Figure 5C). Similar levels of ECAR were measured across treatments after 72 h stimulation, with an increase observed in baseline glycolysis in untrained differentiating macrophages. However, although unchanged after 24 h treatment, we found significant up-regulation in oxidative consumption rates (OCR, an indicator of glucose-dependent oxidative phosphorylation) in BMDM treated with WMP and IVD-WMP after 72 h (Figure 5D). This is consistent with recent reports that β-glucan training employs reprogramming of both glycolysis and TCA to fuel the epigenetic changes required for altered responsiveness (17, 45). Accordingly, targeted inhibition of histone demethylases using 5′methylthioadenosine (MTA) pre-treatment prior to WMP stimulation, blocked the enhanced responsiveness to LPS-restimulation in BMDM (Figure 5E).

### Pre-treatment of bone-marrow cells with mushroom powders drives long-term responses to TLR2 restimulation

**Figure 6:**
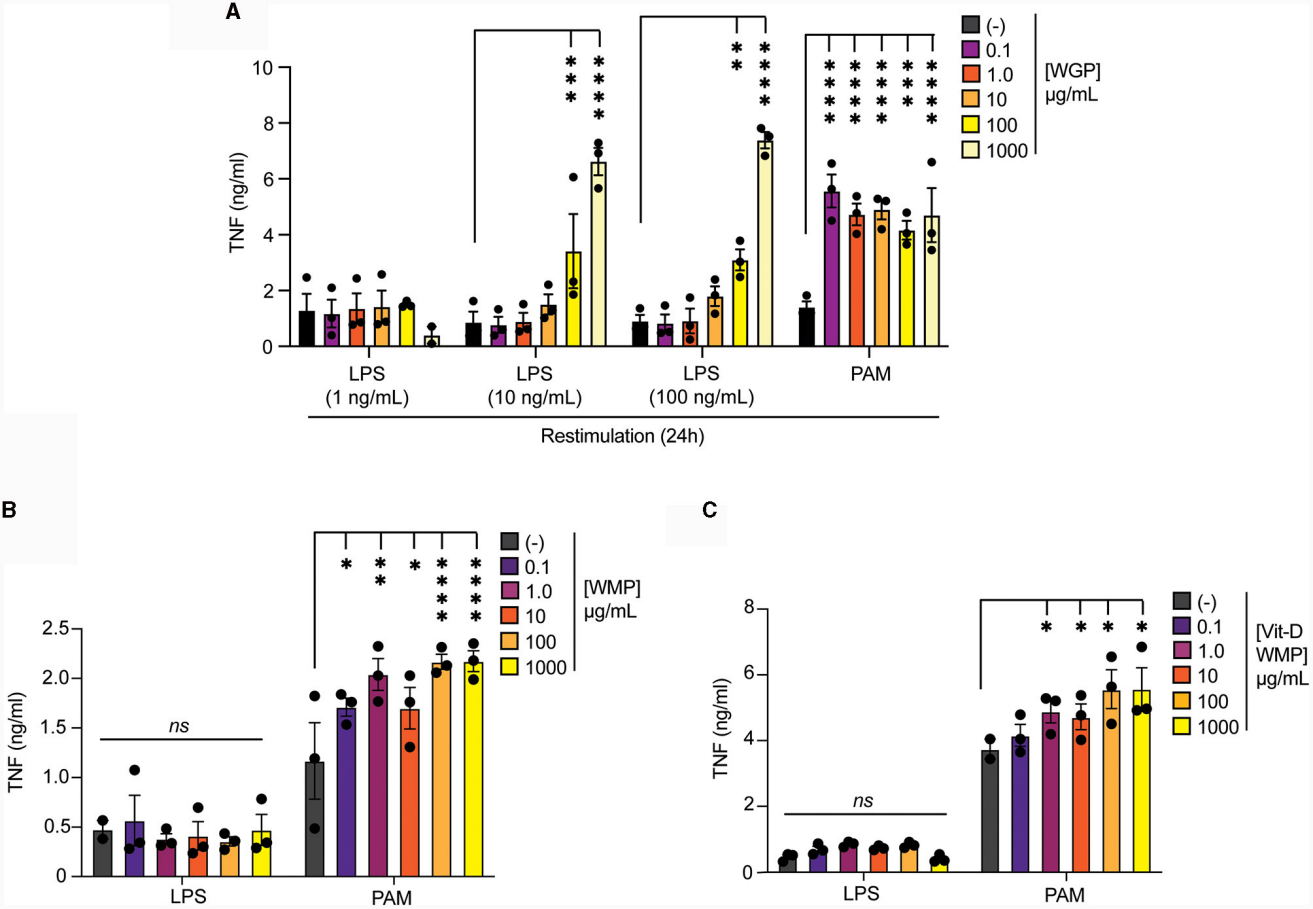
Pre-treatment of bone-marrow cells with mushroom powders drives long-term responses to TLR2 restimulation. **(A–C)** Mature BMDMs were treated with yeast-derived WGP **(A)**, *A. bisporus* derived WMP **(B)**, Vit-A-enriched WMP **(C)** at the indicated concentrations (μg/mL) or left untreated (-). After 24 h, media was removed and cells washed and matured for a further 7 days prior to restimulation with LPS (as indicated or at 10 ng/mL) or Pam3CSK4 (PAM, 10 μg/mL) for 24 h. Supernatant was removed and TNF production measured by ELISA. Data is mean cytokine concentration ± s.e.m. of *n* = 3 independent experiments. *P value < 0.05, **P value < 0.01, ^***^P value < 0.005, ^****^P value < 0.001, *n.s*. *P* value > 0.05, determined using 2-way ANOVA with the indicated *post-hoc* multiple comparisons (Fisher's LSD test).

Intraperitoneal delivery of *Trametes versicolor* β-glucan was shown to alter the frequency and function of myeloid progenitors in bone-marrow (46). Therefore, we examined whether *ex vivo* culture of bone-marrow cells (BMC) with WMP could alter the fate of mature macrophages derived from these cultures. First, we validated this system using yeast-derived WGP. Culture of BMC with WGP for 24 h followed by washing & 5-day maturation, led to enhanced responses to PAM and higher concentration of LPS restimulation with 100–1,000 μg/mL WGP (Figure 6A). Similar culture of BMCs with WMP led to enhanced PAM restimulation responses at most concentrations used (from 1–1,000 μg/mL, Figure 6B), an effect also observed with Vit-D enriched WMP (Figure 6C). Notably, LPS responses were not enhanced by WMP culture, although we noted that the control level of TNF production after LPS treatment was much lower in these cells. Overall, these results suggest that culture of bone-marrow progenitor cells with WMP can enhance the function of mature macrophages derived from HSPCs in these cultures. Whether this represents a direct effect of WMP on HSPCs or other stromal cells is currently unclear.

### Oral delivery of mushroom powder alters bone-marrow cell function and fate

**Figure 7:**
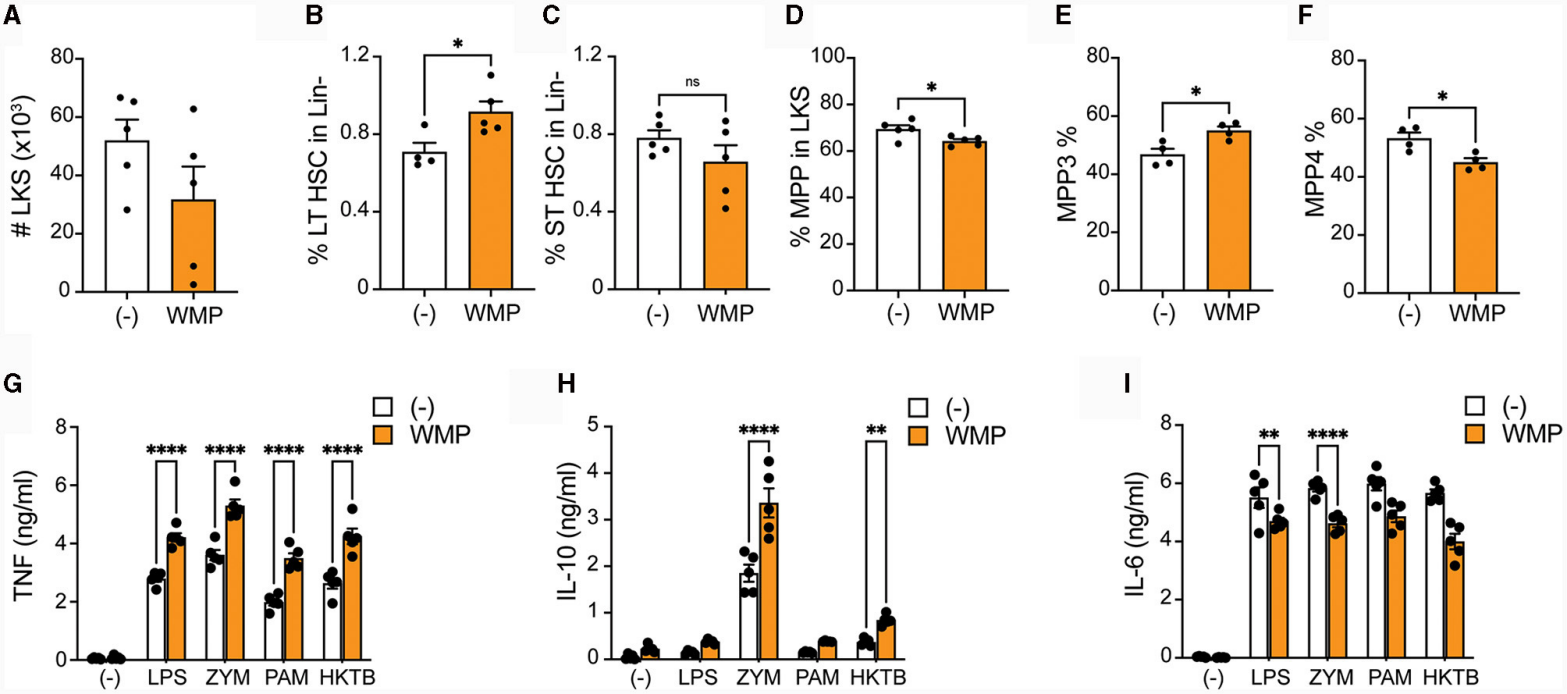
Oral delivery of mushroom powder alters bone-marrow cell function and fate. *A. bisporus*-derived WMP (10 mg) was delivered to C57/BL6 mice via oral gavage daily for 7-days prior to sacrifice. Control mice received PBS (-). Bone-marrow cells were isolated and stained for multi-parameter flow cytometry **(A–F)** or used to generate mature **(G–I)**. **(A–F)** Lineage-, c-kit+, and Sla-1 negative (LKS)-cells were enriched and analyzed by flow cytometry. Graphs shown illustrate differences in absolute cell numbers per leg or relative % differences in populations across PBS and WMP-treated mice. **(G–I)** Mature BMDM were stimulated with LPS (10 ng/mL), Zymosan-A (ZYM, X ug/mL), Pam3CSK4 (PAM, 10 ug/mL), or heat-killed *Mycobacterium tuberculosis* (HKMTB, dose) for 24 h. Supernatant was removed and the indicated cytokines measured by ELISA [**(A)**; TNF, **(B)**; Il10, **(C)**; IL6]. Data is mean cytokine concentration ± s.e.m. of *n* = 5 mice per group. *P value < 0.05, **P value < 0.01, ^****^P value < 0.001, *n.s*. *P* value > 0.05, determined using Students *t*-test.

To more directly address if WMP impacts myelopoiesis, WMP was delivered to mice daily by oral gavage for 1-week prior to sacrifice. Bone-marrow was isolated and HSPCs monitored by flow cytometry. Although the overall number of Lineage-, c-Kit+ and Sca1+ (LKS)-cells was not significantly changed by oral gavage of WMP relative to control PBS-treated mice (Figure 7A), some alterations in the relative frequency of specific subsets was observed. This suggests dynamic remodeling of bone-marrow HSPC populations. Notably, long-term (LT)-HSPC's were increased after WMP delivery, while corresponding short-term (ST)-HSPCs were not changed (Figures 7B–D). The more-committed multi-potent progenitors (MPPs) were not significantly altered by WMP delivery either (Figure 7D). However, examining the specific lineages within these we found an increased proportion of the myeloid-committed MPP3 population in WMP-treated mice, with a corresponding decrease in the proportion of lymphoid-committed MPP4 cells (Figures 7E, F). This skew toward myelopoiesis is characteristic of *in-vivo* Trained Immunity (47), therefore we tested the function of BMDM derived from the same bone-marrow. Consistent with increased activity, production of TNF induced by a range of macrophage activating stimuli was enhanced in WMP-treated mice, from LPS, PAM but also seen in response to ZYM and heat-killed *Mycobacterium tuberculosis* (hk-MTB, Figure 7G). A similar enhancement in IL-10 production was observed (Figure 7H). Curiously, IL-6 production was decreased in BMDM from WMP-treated mice (Figure 7I). Despite this, the increased TNF production and expansion of myeloid progenitors in mice receiving WMP suggests the ability of orally delivered WMP β-glucan to drive Trained Immunity *in-vivo*.

### Chemical analysis of *A. bisporus* mushroom powder and it's *in vitro* digested product

**Figure 8:**
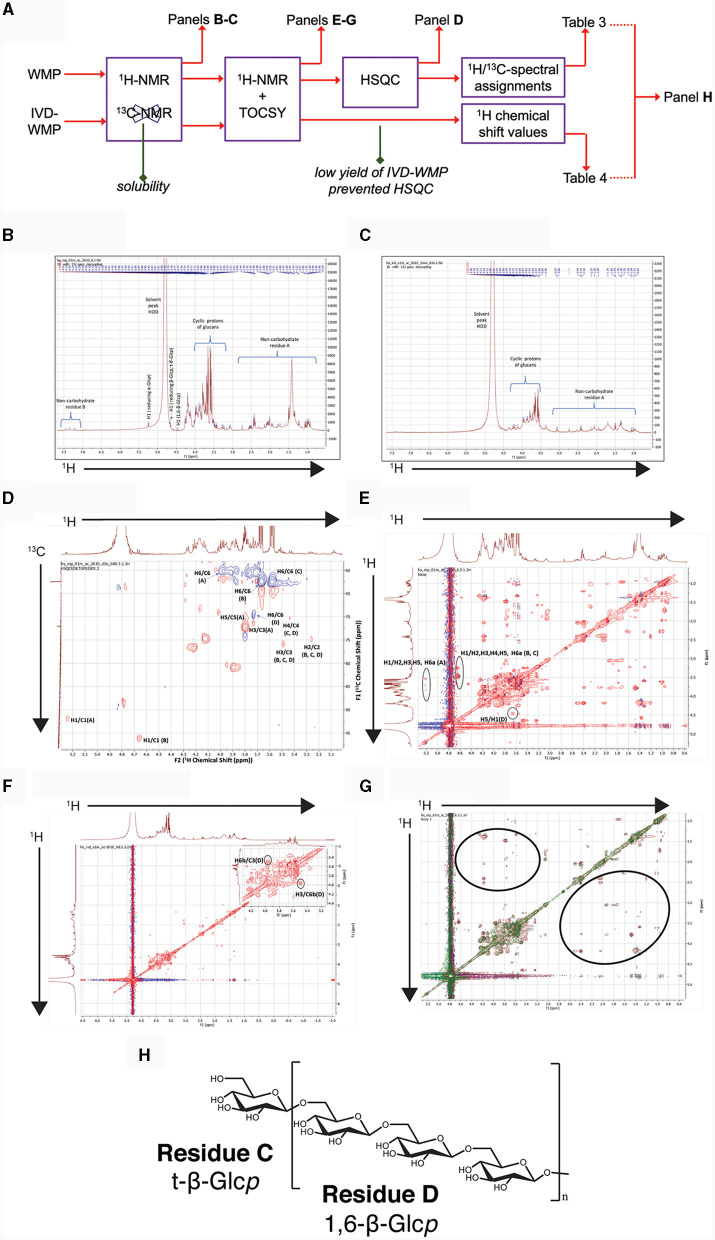
Chemical profiling of mushroom powder and IVD-WMP. **(A)** Overview of workflow used for both *A. bisporus* WMP and digested product (IVD-WMP) showing how subsequent plots were derived. **(B, C)** ^1^HNMR spectrum of WMP **(B)** and IVD-WMP **(C)** dissolved in deuterated water (99.95%) as solvent, with the solvent peak at 4.80 ppm used as a reference peak. Signals in aliphatic (non-carbohydrate residue **(A)** and aromatic (non-carbohydrate residue **(B)** regions are indicated, alongside anomeric protons of glucans. **(D)** 2D-NMR HSQC spectrum of WMP; Residues **(A–D)** represent reducing α-Glc*p*, reducing β-Glc*p*, t-β-Glc*p*, and 1, 6-β-Glc*p*, respectively. **(E)** TOCSY spectrum of WMP; **(F)** TOCSY spectrum of IVD-WMP; **(G)** Superimposed TOCSY spectrum of WMP and IVD-WMP, brown and green signals represent WMP and IVD-WMP respectively. Absence of green signals in regions marked by black ring indicates digestion of WMP carbohydrates and enrichment of β-glucans. **(H)** Predicted structure of *A. bisporus* β(1→6)-glucans based on 2D-NMR and annotations made in Table 3.

Our data suggests that β-glucans in *A. bisporus* mushroom powder drive Trained Immunity in myeloid cells and retain this property after digestion. We thus undertook chemical profiling of WMP and IVD-WMP samples using one-dimensional and two-dimensional NMR analysis to detect and characterize *A. bisporus* β-glucans. The workflow is outlined in Figure 8A. Briefly, partial solubility prevented the generation of accurate ^13^C spectra by 1D NMR, although ^1^H NMR spectra were generated and are shown in Figures 8B, C for both WMP and IVD-WMP, respectively. ^1^H NMR of WMP reveals a complex spectrum with several signals, indicating a mixture of metabolites. As *A. bisporus* mushroom has been reported to contain carbohydrates (glucans), lipids and amino acids as major constituents (33, 48, 49) and our study focuses on immunomodulatory effects of glucans, the spectrum shown is labeled for glucans (middle) and non-carbohydrate residues A & B (to the left and right of glucans). Residue A region ranges from 0.93–2.75 ppm and B ranges from 6.9–7.5 ppm, which covers aliphatic and aromatic protons respectively, that might correspond to lipids and amino acids (49, 50). The region between 3.0 ppm and 4.0 ppm with relatively high peak abundance denotes adequate amounts of glucans as discerned by the cyclic protons of glucosyl moieties. Though a sharp signal occurs at δ1.36 in the aliphatic region, it corresponds neither to methyl group at C6 of deoxy sugars [fucose (δ1.26) or rhamnose (δ1.18)] (51, 52), nor represents -OCH_3_ group as in 3-*O*-methylgalactose (δ3.45), a moiety commonly produced by oyster mushroom, *Pleurotus citrinopileatus* (53).

To further characterize the carbohydrates present, ^1^H NMR (Figures 8B, C) and TOCSY (Total Correlation Spectroscopy; Figures 8E–G) measurements were made on a 400 MHz NMR instrument to capture maximum chemical information about WMP and IVD-WMP. Measurements at 600 MHz NMR were attempted to acquire ^13^C NMR spectra; however, this was only achievable for WMP. The digestion process which alters total carbohydrate content by enriching for fibers (Table 2) impacts solubility, leading to a lower ^13^C signal abundance for IVD-WMP. For this reason, HSQC (Heteronuclear Single Quantum Coherence) of WMP sample only was recorded on 600 MHz instrument (Figure 8D). Consequently, ^1^H/^13^C spectral assignments were obtained for WMP (Table 3), while for IVD-WMP interpretation was limited to ^1^H chemical shift values (Table 4). Due to the complexity of signals generated, we have primarily focused on assigning chemical shifts corresponding to glucans (Tables 3, 4). The signals at δ5.24 and δ4.66 correspond to anomeric protons of reducing α-glucopyranosyl (α-Glc*p*, **A**) and β-glucopyranosyl (β-Glc*p*, B) residues, while signals at δ4.66 and δ4.46 correspond to anomeric protons of terminal β-Glc*p* (**C**) and (1→6)-β-Glc*p* (**D**) residues respectively. This assignment was complicated due to the absence of ^13^C NMR spectrum. Hence for WMP, the ^1^H/^13^C assignments were first picked by HSQC data and then correlated with TOCSY data. Since IVD-WMP was obtained by digesting WMP, their ^1^H NMR and TOCSY spectra were compared (Figures 8B, C, G) and ^1^H chemical shift values were deduced. For residues **A**, chemical shifts corresponding to H1/C1, H2/C2, H3/C3, H5/C5, and H6a,b/C6 were assigned as 5.24/91.83, 3.57/70.31, 3.74/71.18, 3.80/70.45, and 3.71, 3.97/60.35 respectively. The signals for H4/C4 were not found. Similarly, the signals for residues **B** corresponding to H1/C1, H2/C2, H3/C3, H4/C4, H5/C5, and H6a,b/C6 were assigned as 4.66/96.08, 3.26/74.85, 3.48/75.87, 3.41/-, 3.50/-, 3.68, and 3.87/62.40, respectively. For this moiety, δC4 and δC5 were not found in HSQC and hence δH4 and δH5 were assigned based on TOCSY correlations. Similarly, the chemical shifts corresponding to anomeric signals for residues **C** (δ4.66) and **D** (δ4.46) were not found in HSQC but deduced from TOCSY interactions. The residues **C** and **D** have the same shift values for H2/C2 (3.26/74.85), H3/C3 (3.48/75.87), and H4/C4 (3.44/70.31) but differ for H5 (δ3.50 for **C**; δ3.55 for **D**) and H6a,b/C6 (3.58, 3.87/62.40 for **C**; 3.73, 3.97/69.72 for **D**), due to the linkage at C6 in residue **D**, thereby shifting the proton and ^13^C signals downfield. For IVD-WMP, the signal intensity of ^1^H NMR spectra was very low compared to WMP as visible in Figures 8B, C, which made the data interpretation challenging. As a result, complete NMR assignment was not possible, but the signals corresponding to H3, H4, H5, and H6a,b have been labeled for the residues **A**, **B**, **C**, and **D** (Table 4). The peak assignment for WMP and IVD-WMP was found to be in accordance with literature (54, 55). In summary, we identified reducing α- and β-Glc*p*, terminal β-Glc*p* and 1, 6-β-Glc*p* residues and assigned δH/δC chemical shifts for WMP and δH for IVD-WMP. The results establish the presence of α-glucose, β-glucose, and (1→6)-linked β-glucans in mushroom powder. Focusing on β-glucans, we used this data to generate a predicted structure of *A. bisporus* powder β-glucans, shown with a terminal glucopyranosyl unit and a repeating unit of β(1→6)-linked glucopyranosyl units (Figure 8H).

**Table 3:** ^1^H and ^13^C NMR spectral assignments based on HSQC and TOCSY data for polysaccharide residues present in mushroom powder (WMP).

**Assignment**	**H1/C1**	**H2/C2**	**H3/C3**	**H4/C4**	**H5/C5**	**H6a, H6b/C6**
‘A'	5.24/91.83	3.57/70.31	3.74/71.18	-	3.80/70.45	3.71, 3.97/60.35
Reducing α-Glc*p*						
TOCSY correlations	3.71(H6a)/5.24(H1)	5.24(H1)/3.57 (H2)	5.24(H1)/3.74 (H3)	-	5.24(H1)/3.80 (H5)	5.24(H1)/3.71(H6a)
		3.74(H3)/3.57 (H2)	3.57 (H2)/3.74 (H3)		3.97(H6b)/3.80 (H5)	3.57 (H2)/3.71(H6a)
						3.80 (H5)/3.97(H6b)
‘B'	4.66/96.08	3.26/74.85	3.48/75.87	3.41/-	3.50/-	3.68, 3.87/62.40
Reducing β-Glc*p*						
TOCSY correlations	3.26(H2)/4.66(H1)	4.66(H1)/3.26(H2)	4.66(H1)/3.48(H3)	4.66(H1)/3.41(H4)	4.66(H1)/3.50(H5)	4.66(H1)/3.68(H6a)
	3.50(H5)/4.66(H1)	3.48(H3)/3.26(H2)	3.26(H2)/3.48(H3)	3.26(H2)/3.41(H4)	3.48(H3)/3.50(H5)	
	3.41(H4)/4.66(H1)	3.41(H4)/3.26(H2)	3.87(H6b)/3.48(H3)			
‘C'	4.66/-	3.26/74.85	3.48/75.87	3.44/70.31	3.50/-	3.58, 3.87/62.40
t-β-Glc*p*						
TOCSY correlations	3.26(H2)/4.66(H1)	4.66(H1)/3.26(H2)	4.66(H1)/3.48(H3)	4.66(H1)/3.44(H4)	4.66(H1)/3.50(H5)	4.66(H1)/3.68(H6a)
	3.50(H5)/4.66(H1)	3.48(H3)/3.26(H2)	3.26(H2)/3.48(H3)	3.26(H2)/3.44(H4)	3.48(H3)/3.50(H5)	3.58(H6a)/3.87(H6b)
	3.48(H3)/4.66(H1)	3.44(H4)/3.26(H2)	3.87(H6b)/3.48(H3)			
	3.44(H4)/4.66(H1)					
‘D'	4.46/-	3.26/74.85	3.48/75.87	3.44/70.31	3.55/-	3.73, 3.97/69.72
1, 6-β-Glc*p*						
TOCSY correlations	3.55(H5)/4.46(H1)	3.48(H3)/3.26(H2)	3.26(H2)/3.48(H3)	3.26(H2)/3.44(H4)	3.48(H3)/3.55(H5)	3.26(H2)/3.73(H6a)
		3.44(H4)/3.26(H2)	3.73(H6a)/3.48(H3)	3.48(H3)/3.44(H4)	3.73(H6a)/3.55(H5)	3.55(H5)/3.73(H6a)
						3.73(H6a)/3.97(H6b)
						3.48(H3)/3.97(H6b)
						3.97(H6b)/3.73(H6a)

**Table 4:** Proton spectral assignments based on ^1^HNMR and TOCSY data for polysaccharide residues present in *in vitro* digested mushroom powder (IVD-WMP).

**Assignment**	**H1**	**H2**	**H3**	**H4**	**H5**	**H6a, H6b**
‘A'	-	3.58	3.72	-	3.79	3.72, 3.96
Reducing α-Glcp						
TOCSY correlations		3.72(H3)/3.58 (H2)	3.58 (H2)/3.72 (H3)	-	3.96(H6b)/3.79 (H5)	3.58 (H2)/3.72(H6a)
					3.58(H2)/3.79 (H5)	3.79 (H5)/3.72(H6a)
						3.79 (H5)/3.96(H6b)
‘B'	-	-	3.49	3.41	3.51	3.68, 3.86
Reducing β-Glcp						
TOCSY correlations	-	-	3.41(H4)/3.49(H3)	-	-	3.86(H6b)/3.68(H6a)
‘C'	-	-	3.49	3.43	3.51	3.58, 3.89
t-β-Glcp						
TOCSY correlations				3.51(H5)/3.43(H4)	3.43(H4)/3.51(H5)	3.58(H6a)/3.89(H6b)
‘D'	-	-	3.49	3.43	3.55	3.79, 3.96
1, 6-β-Glcp						
TOCSY correlations	-	-	3.96(H6b)/3.49(H3)	-	-	3.49(H3)/3.96(H6b)
						3.79(H6a)/3.96(H6b)

In relation to the effect of simulated digestion, ^1^H NMR analysis of IVD-WMP (Figure 8C) shows the absence of anomeric and aromatic signals. Further, the strong peak at δ1.38 was drastically reduced. Although the overall signal intensity was reduced relative to WMP, the ratio of the glucan to non-carbohydrate region A peaks is increased in IVD-WMP, suggesting enrichment of glucans. The TOCSY spectra of WMP and IVD-WMP (Figures 8E, F), along with the superimposition of both TOCSY (Figure 8G), also demonstrates a dramatic effect of digestion. The circled regions display signals indicating non-carbohydrate residues present in WMP which are lost in IVD-WMP, while the complex carbohydrate regions shown by cyclic protons signals of glucans are preserved in IVD-WMP (overlapping regions). All these observations establish that *in vitro* digestion of WMP proved to be effective in degrading other compounds and enriching β-glucans and thus explain why in our in-vitro assays (Figures 3–5) and oral delivery *in-vivo*, digested mushroom powders retain the ability to drive Trained Immunity.

## Discussion

Although powdered mushroom contains lower overall levels of β-glucan, the data herein demonstrates that delivery of powders to mouse and human innate immune cells can drive functional reprogramming to similar levels as that observed with equivalent amounts of more common β-glucan purifications. That this property and the presence of complex β-glycosidic-linked carbohydrates is conserved after simulated digestion of the powders and coupled with features of Trained Immunity observed in mice after oral delivery of large amounts of the powders, supports the notion that mushroom consumption can support overall innate immune function. These findings may explain in-part some of the positive health benefits previously ascribed to dietary mushrooms or mushroom-derived functional foods, including anti-cancer, anti-oxidant effects, gut health supports and immunomodulatory roles (56–60).

Heterogeneity in β-glucan chemical structure impacts biological activities (8, 9). Mushroom β-glucans are thought to be more linear, β(1→3) linked polymers, with less β(1→6) branching than seen in yeasts and other fungi (eg; *Aspergillus*) (29, 61). As a result of shorter chains, their reported MW is lower (seen in Lentinan and Pleuran β-glucans) and this can affect their solubility (62). Our NMR analysis of *A. bisporus*, although limited, detected predominantly β(1→6) linked glucans with little evidence for branching, although this cannot be discounted. The presence of these β(1→6) linkages confers recognition by Dectin-1 (6) and could be crucial for the induction of Trained Immunity by *A. bisporus* products. Although mushroom β-glucans do not contain long, linear β(1→4) and β(1→3) linkages seen in plants (cereals) and algae (e.g., Laminarin) (63, 64), the binding properties for different Dectin-1 isoforms displayed by *A. bisporus* WMPs reported here, suggests similar biological activity (13). Low MW β-glucans are known to antagonize binding to Dectin-1 by larger particulates (12, 14) and have also been reported to have anti-inflammatory properties (36) similar to that reported for *A. bisporus* powders by us and others (35, 65). Despite this, there are reports of larger MW β-glucans, with increased degrees of branching, across different mushroom species (e.g., Schizophylum and Cordyceps) (29, 61) and these may act differently to *A. bisporus* WMP's reported here. A caveat to interpretation of our data is that for *in-vitro* work with immune cells, our WMPs are filtered. This reduces total carbohydrate content (Table 2) and may preclude larger MW β-glucans normally found in edible mushrooms from our *in-vitro* experiments. Thus, our results likely reflect the function of lower MW *A. bisporus* β-glucans and other smaller, less complex polysaccharides (62), as detected by our NMR analysis. Similarly, our *in-vivo* delivery of WMP used WMPs after filtration, to remove potential contamination of endotoxins which may impact immune function *in-vivo*.

As well as using 1D and 2D NMR chemical analysis to characterize the glucan component of *A. bisporus* WMP's and thereby explain differences in biological activities relative to other β-glucan's tested, this also allowed us to determine the effect of simulated digestion of mushroom products (IVD-WMP). Despite the challenge of obtaining clean ^13^C NMR spectra for the two samples due to their low solubility in deuterated water (D_2_O), ^1^H, HSQC, and TOCSY played a significant role in identifying reducing α- and β- Glc*p*, terminal β-Glc*p* and 1, 6-β-Glc*p* residues and assigning δH/δC chemical shifts for WMP and δH for IVD-WMP. The results establish the presence of α-glucose, β-glucose and β(1→6)-linked glucans in mushroom powder. There have been several reports on α- and β-glucans from *Agaricus bisporus* (33, 66–68), present as a backbone, with other monosaccharides (galactose, fucose, xylose, and mannose) attached as side chain or independently in small amounts (33). We came across only one report on fucogalactan isolated from *A. bisporus* with a (1→6)-linked α-Galp backbone and fucose branching (52). Although more commonly used for storage and energy utilization, less is known about the immunomodulatory effects of α-glucans. Though present in much lower amounts than β-glucans in our *A. bisporus* mushroom powders, there was a striking increase in α-glucans composition in powders from Selenium enriched WMPs, which did not display the ability to drive trained responses in mouse macrophages. It remains to be seen if their increased abundance simply outcompetes mushroom β-glucans or if α-glucans can directly block Dectin-1 binding. The presence of α-glucose, as well as β(1→6) linked glucans was inferred in digested WMP, while other simpler carbohydrate and non-carbohydrate residues seemed to be lost. Although the signal obtained was lower, which impeded complete assignment of residues in IVD-WMP, our results suggest that the simulated digestion process modifies the carbohydrate content and abundance of WMPs. Although the overall amount likely decreases as a result of digestion, the relative abundance of complex, glucan species compared to simpler carbohydrates increases. Thus, digestion *in-vivo* likely preserves dietary fibers like β-glucans which can then mediate biological and immune effects.

In support of this, our *in-vivo* delivery of filtered WMP via oral gavage led to features of Trained Immunity, including bone marrow myelopoiesis and enhanced responses to stimulation in BMDM from trained mice. Many groups have observed expansion in the total frequency and amount of bone marrow hematopoietic stem and progenitor cells (HSPCs), defined as LKS+ cells, after *in-vivo* delivery of training stimuli, generally via intra-peritoneal injection (21, 46). Other studies of oral β-glucan delivery in humans did not find evidence of trained responses in circulating PBMCs (69), which suggests that the route of administration is central to the development of trained responses. Previous work in our lab (*in-press*) revealed that although IP injection of yeast β-glucans leads to a similar expansion in LKS+ cells, oral gavage of equivalent amounts of the same β-glucan did not alter total LKS+ numbers, similar to what was observed here after WMP gavage. Despite this, in both cases—after yeast β-glucan and WMP gavage, the populations of more committed hematopoietic progenitor cells (HSCs), particularly the multi-potent progenitors (MPPs) revealed a skew toward myeloid progenitors (MPP3), indicative of increased myelopoiesis. We hypothesize that oral delivery can indeed reprogramme myeloid progenitors, as evidenced herein by enhanced functional responses in mouse BMDM. However, this route of administration likely avoids the typical systemic inflammatory response seen with peritoneal delivery, which may contribute overall to the inflammatory milieu suggested to be required to trigger emergency granulopoiesis (70) and thus give rise to the noted expansion in total LKS+ cells after delivery. How these 2 processes may be disentangled in response to specific stimuli and routes of administration and their relative contributions to features of central Trained Immunity remain unclear. Although direct incubation of bone-marrow cells with WMP *in-vitro* led to some trained responses, this population contained both HSPCs and mature bone-marrow resident cells which may mediate the training effect. This, coupled with the lack of expansion in total LKS+ cells after oral delivery, suggests a more indirect route mediating *in-vivo* Trained Immunity after oral delivery of WMPs.

The short-term delivery of β-glucans and WMP employed here precludes a role for major changes in the architecture of gut microbiome and their metabolites (71), although this cannot be completely discounted. Although mammals are not thought to express β-glucanases, previous experiments have demonstrated that some soluble β-glucans are absorbed after oral delivery including laminarin and glucan-phosphate (72). Importantly, these treatments were associated with increased non-specific immune responses (72), analogous to Trained Immunity. While a role for the gut microbial flora as a source of β-glucanases cannot be discounted, our simulated digestion modified β-glucan composition of WMP and preserved the capacity to train macrophages *in-vitro*, independently of the microbiome. Thus, WMP-derived products likely mediate the features of Trained Immunity observed *in-vivo*, although their direct substrate remains unclear. Analysis of RNA and proteomic sequencing databases (e.g., The Human Protein Atlas) and experimental evidence demonstrates functional expression of both Dectin-1(a/b) isoforms in primary human intestinal epithelial cells, which regulate biological responses to β-glucan treatment (73), including cytokine production. Other work has demonstrated that macrophages can phagocytose larger, insoluble β-glucan particulates to release soluble β-glucans and thereby mediate their biological effects (74). Further work will determine if intestinal recognition of dietary β-glucans—either intact particulates or more soluble products of digestion—occurs through epithelial or gut-associated lymphoid tissue (GALT)-mediated Dectin-1 expression and if this mediates Trained Immunity via the bone-marrow, possibly through regulating systemic cytokine responses or other mechanisms.

Our model of *in-vivo* WMP delivery mimicked daily consumption of common white button mushroom containing foods. Analysis after 1 week revealed features of Trained Immunity. However, the longevity of innate immune memory triggered through this pathway remains unclear. Indeed, whether repeated dosing functions similarly to repeat prime/boost immunization strategies or reaches a threshold level—after which tolerance mechanisms or negative regulation kicks in, is currently unclear. Studies of innate memory triggered by the endotoxin component LPS suggest that lower concentrations of stimuli trigger Trained Immunity (75, 76), while large amounts trigger a tolerance phenotype (44). The modest trained response measured here with dietary WMP dosing may be sufficient to augment innate immunity, yet avoid negative regulation feedback despite repeated dosing. At the same time, Trained Immunity has now been described as underlying pathogenesis in many inflammatory diseases including diabetes and arthritis (77, 78), with triggering of innate immune cells by damage-associated molecular patterns (DAMPs) preprogramming for increased activity in disease (3, 79). We hypothesize that training with β-glucans, although it enhances pro-inflammatory cytokine production via epigenetic priming, does so in a non-specific fashion. Although anti-inflammatory cytokines like IL10 have not been reported to be enhanced by β-glucan training (5), their expression is not lost—consistent with a self-limiting nature to the trained innate immune response. Indeed, in our *in-vivo* WMP model increased IL10 was observed. In this way, strategies which target Trained Immunity will not skew toward a generalized pro-inflammatory response, but rather promote enhanced, balanced responses. In this way, Trained Immunity increases early responses to infection, which if delayed, can lead to dissemination of infection and uncontrolled inflammation -exemplified by defects in early viral containment associated with severe COVID-19 (80). Similarly, particulate β-glucans have been shown to restore defective inflammatory responses in models of chronic wound healing (81) and Trained Immunity may underlie the augmented macrophage response observed. Dietary manipulation of innate immune function through the delivery of modest amounts of β-glucan containing foods thus, may in fact represent a beneficial way to fortify the immune system. As well as being traditionally linked with health benefits, and more recently investigated for anti-cancer, anti-inflammatory and anti-oxidant properties (26, 34, 60), mushroom products—specifically the powdered *A. bisporus* products employed here—have been associated with increased animal health—with measurable changes in body weight and lifespan (37). Whether Trained Immunity underlies these changes remains unclear, but warrants future investigation.

In summary, we have demonstrated that powdered products of the common and edible white button mushroom *A. bisporus*, contain β-glucans with the capacity to drive Trained Immunity in innate immune cells *in-vitro*. This powdered form mimics the products of mastication, however simulated digestion preserves this property. Although the overall carbohydrate content is altered by simulated digestion, our NMR analysis suggests that β-glucans are retained. Oral administration of powdered *A. bisporus* products leads to features of Trained Immunity in mouse bone-marrow and derived mature macrophages. The approaches employed here provide both a rationale and mechanism to investigate further whether reprogramming of innate immune cells by mushrooms products enhances effector functions in these circumstances.

## Methods

### Mushroom powders and fractionation

All powders used in this study were from *A. bisporus* mushroom crops obtained from MBio, part of the Monaghan Mushrooms group as previously described (37). Powders were re-suspended in 1x PBS for cell culture and animal experiments and incubated overnight rolling at 37°C to dissolve. Solutions were then passed through 20 μM filters and aliquoted for use. For fractionation studies, *A. bisporus* mushroom stalk powder (25 g) was suspended in deionised water (250 mL) and stirred at room temperature for 6 h. Supernatant (Cold Water extract, CW-E) was prepared using centrifugation. The residue from CW-E was re-suspended in water and heated to 60°C for 6 h. Supernatant (Hot Water extract, HW-E) was prepared using centrifugation. Thermal concentration of the HW-E supernatant following three consecutive hot water extractions was condensed by boiling and precipitated by diluting it 1:3 in ethanol. The residue from three hot water extractions was suspended in 1M KOH and heated to 60°C for 20 min. The pH of the extract was adjusted to 7.4 using HCl. Supernatant (KOH-E) was prepared using centrifugation. Supernatant was condensed by boiling. The pH of the extract was adjusted to 7.4 using HCl. The residue from the 1M KOH extraction was initially subjected to an acid hydrolysis using NaNO_2_ and HCl to break linkages with chitin. The alkali-soluble “free” glucans were captured using 1M NaOH. The pH of the extract was adjusted to 7.4 using HCl (NaOH-E). Extracts were weighed and diluted from 1:1,000 to 1:10 for cell assays.

### Simulated *in-vitro* digestion of mushroom powder

Simulated oral, stomach and intestinal digestion fluids were prepared as buffers as outlined in the published INFOGEST protocol (39). Enzyme activity for each digestive enzyme preparation (pepsin, gastric lipase, and pancreatin) was measured and concentrations used adapted according to Minekus et al. (82) such that pepsin (2,000 U/mL), gastric lipase (60 U/mL) and pancreatin measured by trypsin activity was 100 U/mL. One g of mushroom powder was dissolved in simulated oral digestion fluid for 2 h at 37°C without enzymatic digestion, since the mincing procedure mimics mastication. Gastric digestion fluid, buffer and enzymes were added to the oral bolus and digested for a further 2 h at 37°C with shaking. One hour prior to intestinal digestion, bile was added to solubilise. Pancreatin was prepared and the intestinal digestion carried out for 2 h at 37°C with shaking. After digestion, mushroom products were centrifuged at 200 rcf for 5 min. Pelleted residue was collected (*in-vitro* digested mushroom product) and subject to dialysis in deionised water (3–4 water changes). The content from dialysis bags was moved aseptically into petri dishes and subjected to freeze drying at −80°C resulting in the product referred to as *A. bisporus in-vitro* digested whole mushroom powder (IVD-WMP). For cell experiments, IVD-WMP powder was prepared similarly to WMPs described above, by resuspension in PBS, overnight shaking incubation, and filter sterilizing prior to use.

### Cell stimulations

All PRR ligands (*E.coli*, ultrapure lipopolysaccharide, yeast Zymosan-A, Pam3CSK4, *L. digitata*-derived Laminarin, and heat-killed) *Mycobacterium tuberculosis* were purchased from Invitrogen. Yeast whole-glucan particle was a gift to FJS from Kerry Group (Ireland).

### β-glucan and total carbohydrate quantification

β-glucan content of whole mushroom powder and IVD-WMP was determined using the β-Glucan Assay Kit (Yeast and Mushroom, Product code: K-YBGL). Briefly, total glucan content was measured by solubilizing all glucans after acid denaturation and digesting and oxidizing glucose units. α-glucans were measured after alkali hydrolysis with subsequent glucose oxidation and this value was subtracted from a value obtained for total soluble glucans obtained after acid denaturation, digestion and glucose oxidation. The *Mega-Calc*™ software tool was used for raw data processing and analysis.

Carbohydrate content of WMPs was measured using a resorcinol sulphuric acid method, based on a reported protocol (83). Briefly, 25 μL of sample solutions (250 μg/mL, 500 μg/mL) were pipetted in 96-well microtiter plate, to which 25 μL of a freshly prepared resorcinol solution (10 mg/mL) was added followed by addition of 105 μL of concentrated sulfuric acid with vigorous mixing. The plate was heated for 30 min at 90°C in oven and then cooled to room temp. in dark with regular shaking for another 30 min. Finally, the absorption of the resulting brownish orange color was measured by a BMG microtiter plate reader at λ = 450 nm, with λ = 690 nm as a reference wavelength. A calibration curve generated from glucose standards (conc. 10–1,000 μg/mL) was used to quantify the carbohydrate content.

### Reporter cell line assays

HEK-Blue hDectin-1a and HEK-Blue hDectin1b (3–7 × 10^6^ cells) were cultured in growth medium (GlutaMAX™ DMEM, 10% FBS, 50 μg/ml Penicillin/Streptomycin, 100 μg/ml Normocin) with the addition of selective antibiotics (Puromycin, HEK-Blue™ CLR Selection). For reporter assays, cells were plated in flat-bottomed 96 well plates at 1 × 10^5^ cells/well in 180 μL HEK growth medium, minus selective antibiotics. Cells were treated with 20 μL of PRR ligands/mushroom solutions and incubated at 37°C for 24 h. Cell supernatants were harvested for QuantiBlue SEAP quantification. SEAP activity was assessed by reading the optical density (OD) at 595 nm with a microplate reader.

### Mouse bone-marrow derived macrophages culture and training assays

BMDM were isolated as described (84). After isolation and red blood cell lysis (Lysis Buffer Hybri-Max^TM^, Sigma-Aldrich), bone marrow cells were resuspended in DMEM, 10% FBS, 20% L929-conditioned media and seeded to be differentiated into BMDMs. Five days after isolation, mature BMDM were lifted by placing on ice and reseeded at the required density (1 × 10^6^ cell/mL) in DMEM, 10% FBS, 20% L929-conditioned media and allowed to rest overnight. Six-days post isolation, re-seeded mature BMDM were incubated with training stimuli (WGP or WMPs) for 24 h. Seven-days post-isolation, media was removed and cells washed three times and incubated in fresh media (DMEM, 10% FBS, 5% L929-conditioned media) for a further 5 days, changing the media after 3 days. Six-days post training, BMDM were stimulated with PRR agonists and supernatant sampled at 3 h, 6 h, and 24 h time points for analysis of cytokine production. For inhibition experiments, 5′methylthioadenosine (MTA, 1 mM) was added to mature BMDM 2 h prior to addition of WMP. Control cells were treated with a similar volume of vehicle (DMSO/media).

### Human monocyte derived macrophages and training assays

Buffy packs from human blood donations were obtained from the Irish Blood Transfusion Service, St James' Hospital under clinical indemnity. PBMCs were isolated using Lymphoprep and monocytes enriched by centrifugation through a percol gradient (Sigma-Aldrich), as described (18). Monocytes were seeded in 48-well plates at a density of 6 × 10^5^ cells/mL in a volume of 500 μL per well and cultured in RMPI growth media supplemented with 10% human serum (Sigma-Aldrich). Twenty Four hours post isolation the cells were stimulated with training stimuli (WGP/WMPs). After a further 24 h, cells were washed again three times with 1X PBS (+Mg, + Ca) and fresh media was added. On day 6 post isolation, the hMDM's were stimulated with PRR ligands as described. Samples of the supernatants were taken at 3 h, 6 h, and 24 h time points for analysis of cytokine production.

### Cytokine quantification

Supernatant cytokine concentration was determined using Invitrogen Uncoated ELISA kits (Thermo-Fisher) for mouse TNF (# 88-7324-88), IL-6 (#88-7064-77), and IL-10 (# 88-7105-88) as per manufacturer's instructions. After the final incubation with streptavidin-horseradish peroxidase conjugates, plates were washed seven times. Fifty μL of TMB substrate reagent was added to each well. The reaction was stopped with 25 μL of 1 M H_2_SO_4_. The plate was read using a microplate reader set to a wavelength of 450 nm. Microsoft Excel software was used to generate a standard curve from which the cytokine concentration of the samples was determined.

### Metabolic analysis

For metabolic analysis of trained cells, lactate concentration was measured in supernatants using the colorimetric Lactate Assay Kit (MAK064, Sigma-Aldrich). Extracellular flux analyses were carried out using an XFe24 Extracellular Flux analyser (Seahorse Biosciences) in Seahorse Media freshly supplemented with 10 mM glucose and 2 mM l-glutamine. An adapted version of the XF cell mito-stress test was used to measure key parameters of both mitochondrial and non-mitochondrial function through the oxygen consumption rate (OCR) as well as analysis of the extracellular acidification rate (ECAR) of the media to investigate glycolytic flux. Cells were plated in the seahorse plate at 100, 000 cells per well for 24 h stimulation assays or 50,000 cells per well for 72 h stimulation assay. Cells were stimulated as previously described. For 72 h measurements, cells were washed with PBS and 200 uL after 24 h of stimulation and fresh cDMEM/5%LCM was added. On the day prior to measurement the calibration cartridge was hydrated with 200 μL of XF Seahorse calibration media and was placed in a non-CO2 incubator overnight at 37°C. On the day of the assay, cells were washed X2 with seahorse medium. Each well was then topped up with 180 μL of seahorse medium and the plate was placed into a non-CO2 incubator at 37°C for 20 min before the beginning of the Seahorse run. Following calibration and the cell culture plate was loaded for real-time analysis. During the run, the following inhibitors (diluted in seahorse media) were injection to interrogate metabolism oligomycin (2 μM), fluoro-carbonyl cyanide phenylhdrazone (FCCP, 1 μM), rotenone/antimycin-A (0.1 μM/4 μM), and 2DG (30 μM). Normalization for cell number was carried out with a Crystal Violet dye assay.

### Oral delivery of WMP

For *in-vivo* WMP delivery, an oral bolus equivalent to 500 mg of WMP per kg mouse body weight was delivered resuspended in PBS in a maximum volume of 100 μL. Five hundred mg/kg equates to 10 mg WMP in a 20 g adult mouse. The equivalent human dose is 40 g in an 80 kg adult. Mice were given WMP oral gavage bolus once a day at morning-time for 7 days prior to sacrifice. Oral gavage was chosen as the optimal way to deliver the same amount of WMP to all mice via the gastro-oral route. Control mice received PBS. Bone-marrow cells were isolated and used to generate mature bone marrow derived macrophages. Mature BMDMs were stimulated on day 5 post isolation with LPS (10 ng/mL), Zymosan-A (ZYM, 10 ug/mL), Pam3CSK4 (PAM, 10 ug/mL) or heat-killed *Mycobacterium tuberculosis* (HKMTB, MOI 5) for 24 h. Supernatant was removed and the indicated cytokines measured by ELISA.

### Multiparameter flow cytometry analysis of mouse bone marrow cells

To analyse HSPC populations in mouse bone marrow, HSPC cells were enriched using selection for c-Kit before immune staining and characterization flow cytometry. Isolated bone marrow cells from 1 femur per mouse were centrifuged and resuspended in 125μl of cold PBS. Twenty five μl of anti-CD117 (cKit) beads were added to 20 million bone marrow cells. Cells were resuspended and incubated on ice for 5 min. 1.5 μl of anti-CD117 APC flow antibody was added, gently vortexed and incubated on ice for 15 min in the dark. Five ml of cold PBS was added and cells were centrifuged at 450 G for 5 min at 4°. LS MACs column were activated with the addition of 2 ml of PBS. Cells were resuspended in 1 ml of PBS. Using an insulin syringe and a short 25G needle, cells were transferred onto the LS column. The column was then washed with an additional 1 ml of cold PBS. The column was removed from the magnet and 5 ml of cold PBS was added to the column. cKit positive cells were detached from the column by plunger. cKit+ cells from MACs column were washed with 1 ml PBS. Cells were stained in 100 μl of zombie aqua (Live/Dead stain, concentration 1 in 1,000) for 20 min at 4°. Cells were washed with 1 ml of PBS and centrifuged at 450 G for 5 min. Ten μl of count beads were added to each sample and cells were then incubated with 50 μl of primary antibody master mix (Primary antibody master mix: concentration 1 in 100, biotin conjugated CD11b, B220, CD5, TER119, Ly6G/C, CD8a, and CD4—antibodies made up in PBS) for 30 min at 4°. Cells were then washed with 1 ml of PBS and centrifuged at 450 G for 5 min. Cells were then resuspended in 50 μl of secondary antibody master mix (Secondary antibody master mix: concentration 1 in 100 of CD34 FITC, Flt3 PE, CD48 PerCP-efl710, cKit APC, Streptavidin APC-Cy7, Sca-1 PE-Cy7, and CD150 efl450) for 30 min at 4°. Cells were washed with 1 ml PBS and centrifuged at 450 G for 5 min. Cells were fixed using 100 μl fixation buffer (Fischer Scientific) for 15 min at room temp. Cells were washed with 1 ml PBS and ran on BD FACs Canto. Fluorescence Minus One (FMO) controls were performed using 1 × 10^6^ cells obtained by mixing equivalent volumes of samples coming from the different experimental conditions and stained with the proper antibodies. Compensation controls were obtained after staining UltraComp eBeadsTM Compensation Beads (Invitrogen) with the appropriate antibodies. Cells were acquired on the BD flow cytometer Canto II with FACSDiva software. Data analysis and flow charts were performed using FlowJo software v.7.6 (TreeStar).

### NMR analysis

The homonuclear magnetic resonance experiments (^1^H NMR, TOCSY) were performed on Bruker 400 NMR spectrometer operating at 400.23 MHz, while heteronuclear measurement (HSQC) was performed on Bruker Avance III 600 NMR spectrometer operating at 600.13 MHz and 150.6 MHz. due to low solubility of sample that further reduce the abundance of ^13^C in soluble portion. A 10–15 mg of WMP and IVD-WMP were dissolved in 0.75 mL of deuterated water (99.95%, Sigma-Aldrich) and filtered through prewashed glass wool. ^1^H, HSQC and TOCSY NMR data were recorded at 26°C. For ^1^H NMR, acquisition parameters were as follows: 1,024 scans were recorded with an acquisition time of 2.04 s, relaxation delay of 1 s and spectral width of 8,012.8 Hz. For TOCSY, 128 scans were recorded with an acquisition time of 0.301 s, relaxation delay of 1 s and spectral width of 3,401.4 Hz. For HSQC measurements at 600 MHz instrument, 128 scans were recorded with an acquisition time of 0.095 s, relaxation delay of 1 s and spectral width of 5,411.3 Hz, 22,624.4 Hz. For NMR data processing, all the spectra were referenced to the solvent peak at 4.80 ppm. The spectra were processed and analyzed in MestReNova chemical suite software.

### Data analysis and figure generation

All figures shown represent the mean of independent experiments carried out with a variety of mushroom powder batches manufactured between 2022–2023. For experiments with multiple groups, ANOVA was carried out followed by the indicated *post-hoc* individual tests and *p-*values determined for key comparators as indicated. Raw experimental data for each experiment was compiled and analyzed using Microsoft Excel. GraphPad Prism was used to pool results of replicate experiments and generate graphs. Figures were prepared using Adobe Illustrator.

## Data availability statement

The raw data supporting the conclusions of this article will be made available by the authors, without undue reservation.

## Ethics statement

The studies involving humans were approved by Trinity College Dublin Faculty of STEM Research Ethics Level 2 Committee and Level 1 Sub-Committee. The studies were conducted in accordance with the local legislation and institutional requirements. The participants provided their written informed consent to participate in this study. The animal studies were approved by Trinity College Dublin Animal Research Ethics Committee. The studies were conducted in accordance with the local legislation and institutional requirements.

## Author contributions

SCa: Conceptualization, Data curation, Formal analysis, Investigation, Methodology, Writing—review & editing. TO'B: Data curation, Formal analysis, Investigation, Writing—review & editing. AL: Formal analysis, Investigation, Methodology, Writing—review & editing. SCh: Investigation, Methodology, Writing—review & editing. CH: Investigation, Writing—review & editing. EH: Investigation, Data curation, Formal analysis, Methodology, Writing—review & editing. MO'S: Formal analysis, Investigation, Methodology, Writing—review & editing. HC-M: Investigation, Methodology, Conceptualization, Writing—review & editing. ED: Investigation, Methodology, Writing—review & editing. SY: Investigation, Methodology, Conceptualization, Formal analysis, Project administration, Resources, Supervision, Writing—review & editing. JW: Conceptualization, Formal analysis, Investigation, Methodology, Project administration, Resources, Supervision, Funding acquisition, Validation, Writing—original draft. SCo: Funding acquisition, Methodology, Supervision, Writing—review & editing. SN: Methodology, Conceptualization, Data curation, Formal analysis, Investigation, Project administration, Resources, Writing—original draft. FS: Conceptualization, Data curation, Project administration, Funding acquisition, Supervision, Writing—original draft.
